# Prostanoids in bronchoalveolar lavage fluid do not predict outcome in congenital diaphragmatic hernia patients

**DOI:** 10.1080/09629359791712

**Published:** 1997-06

**Authors:** H. Ijsselstijn, F. J. Zijlstra, J. C. De Jongste, D. Tibboel

**Affiliations:** 1 Department of Paediatric Surgery Erasmus University Rotterdam and University Hospital/Sophia Children's Hospital Rotterdam The Netherlands; 2 Department of Paediatrics Division of Respiratory Medicine Erasmus University Rotterdam and University Hospital/Sophia Children's Hospital Rotterdam The Netherlands; 3 Department of Pharmacology Erasmus University Rotterdam and University Hospital/Sophia Children's Hospital Rotterdam The Netherlands

## Abstract

Vasoactive prostanoids may be involved in persistent pulmonary hypertension (PPH) in infants with a congenital diaphragmatic hernia (CDH). We hypothesized that increased levels of prostanoids in bronchoalveolar lavage (BAL) fluid would predict clinical outcome. We measured the concentrations of 6-keto-prostaglandin F_1α_ (6-keto-PGF_1α_), thromboxane B_2_ (TxB_2_), protein, albumin, total cell count, and elastase-α1-proteinase-inhibitor complex in BAL fluid of 18 CDH patients and of 13 control subjects without PPH. We found different concentrations of prostanoids in BAL fluid of CDH patients with PPH: infants with a poor prognosis had either high levels of both 6-keto-PGF_1α_ and TxB_2_ compared to controls, or high levels of 6-keto-PGF_1α_ only. TxB_2_ levels showed a large variability in all CDH patients irrespective of outcome. We conclude that prostanoid levels in BAL fluid do not predict clinical outcome in CDH patients.


Mediators of Inflammation, 6, 217–224 (1997)

### Prostanoids in bronchoalveolar lavage fluid do not predict outcome in congenital diaphragmatic hernia patients

H. IJsselstijn,[1,2] F. J. Zijlstra,[3] J. C. de Jongste[2] and D. Tibboel[1,CA]

Departments of [1]Paediatric Surgery, [2]Paediatrics, Division of Respiratory Medicine, and [3]Pharmacology, Erasmus University Rotterdam and University Hospital/Sophia Children's Hospital, Rotterdam, The Netherlands

[CA]Corresponding Author
Tel: (+31) 10 4636567
Fax: (+31) 10 4636288
Email: illsley@chis.azr.nl

**Vasoactive prostanoids may be involved in persistent pulmonary hypertension (PPH) in infants with a congenital diaphragmatic hernia (CDH). We hypothesized that increased levels of prostanoids in bronchoalveolar lavage (BAL) fluid would predict clinical outcome. We measured the concentrations of 6-keto-prostaglandin $F_{1\alpha}$ (6-keto-$PGF_{1\alpha}$), thromboxane $B_2$ ($TxB_2$), protein, albumin, total cell count, and elastase-$\alpha_1$-proteinase-inhibitor complex in BAL fluid of 18 CDH patients and of 13 control subjects without PPH. We found different concentrations of prostanoids in BAL fluid of CDH patients with PPH: infants with a poor prognosis had either high levels of both 6-keto-$PGF_{1\alpha}$ and $TxB_2$ compared to controls, or high levels of 6-keto-$PGF_{1\alpha}$ only. $TxB_2$ levels showed a large variability in all CDH patients irrespective of outcome. We conclude that prostanoid levels in BAL fluid do not predict clinical outcome in CDH patients.**

**Key words:** Artificial ventilation, Extracorporeal membrane oxygenation, Congenital diaphragmatic hernia, Persistent pulmonary hypertension, Prostacyclin, Thromboxane

### Introduction

The high mortality of morbidity in children with congenital diaphragmatic hernia (CDH) is largely determined by the severity of lung hypoplasia and persistent pulmonary hypertension (PPH).[1] Despite improved neonatal intensive care, the overall survival rate still does not exceed 60%.[2] Several attempts have been made to predict mortality in these patients. A recent study showed that data reported to the Extracorporeal Life Support Organization Registry of more than 1000 CDH patients treated with extracorporeal membrane oxygenation (ECMO) did not allow discrimination of non-survivors from survivors.[3]

In the lung, arachidonic acid metabolites regulate the bronchial and vascular tone, and are involved in inflammatory processes.[4] Increased levels of eicosanoids have been reported in plasma and in bronchoalveolar lavage (BAL) fluid of children with PPH who were treated with conventional ventilation or with ECMO.[5–8] Increased plasma levels of the stable metabolites of the pulmonary broncho-, and vasoconstrictor thromboxane $A_2$ ($TxA_2$) and the pulmonary vasodilator prostacyclin ($PGI_2$), $TxB_2$, and 6-keto-$PGF_{1\alpha}$ have been observed in CDH patients during episodes of hypoxemia,[9] and in the immediate postoperative period.[10–12] BAL has been used to evaluate different aspects of inflammatory responses, such as the number of neutrophilic granulocytes and macrophages, albumin, elastase, and $\alpha_1$-proteinase inhibitor activity in ventilated preterm infants with respiratory distress syndrome who were likely to develop chronic lung disease.[13–15] We hypothesized that in CDH patents, involvement of vasoactive prostanoids in the pathogenesis of PPH would be reflected by increased prostanoid levels in BAL fluid, and that these concentrations might have a predictive value with respect to clinical outcome.

The aim of the present study was to measure the concentrations of 6-keto-$PGF_{1\alpha}$, $TxB_2$, and various inflammation markers in BAL fluid of CDH patients who were treated either with conventional ventilation or with ECMO and to evaluate the prostanoid levels in relation to outcome. The levels of different parameters in BAL fluid were compared with those of controls without PPH who had similar gestational age and birth weight.

### Patients and Methods

### Patients

The study was performed in our Paediatric Surgical Intensive Care Unit between December

© 1997 Rapid Science Publishers

1993 and January 1996. A group of 18 CDH patients were studied; 13 children underwent conventional ventilation (referred to as the CDH-CV group), and five children were treated with venoarterial ECMO (referred to as the CDH-ECMO group) using standardized treatment protocols.[16–18] Four CDH patients had been diagnosed prenatally. Ten patients in the CDH-CV group and all five ECMO patients suffered from respiratory insufficiency within 6 hours of birth; the other three patients were respiratory insufficient after 10 hours, 36 hours, and 28 days, respectively. Operative repair by an abdominal approach was performed in 11 conventionally ventilated children and in three ECMO patients after preoperative clinical stabilization;[16] four of the five patients who died had not been operated on. All 18 patients routinely received antimicrobial prophylaxis. They underwent echocardiography on admission: right-to-left shunting was diagnosed in six children of the CDH-CV group and in all CDH-ECMO patients.[12] Clinical evidence for right-to-left shunting was obtained by preductal and postductal transcutaneous $O_2$-saturation differences of $> 10\%$ in five CV-patients and in all ECMO patients.

For CDH patients in our institution the entry criteria for ECMO were: gestational age of at least 34 weeks; birth weight at least 2000 grams; artificial ventilation for less than 7 days; alveolar–arterial oxygen difference ($A\text{-}aDO_2$) $> 80$ kPa (600 torr); maximal $PaO_2$ at least 10 kPa. During ECMO ventilatory settings were usually reduced to PIP 12–16 cm $H_2O$, PEEP 5–6 cm $H_2O$, rate 10–15/min, and $FIO_2$ 0.25–0.3.

Thirteen other children without pulmonary abnormalities, who were mainly ventilated perioperatively at a median age of 2.5 days (range 1–8 days), served as age-matched controls. They were selected for the best possible match for age, gestational age and birth weight. They all received antimicrobial therapy. Echocardiography was performed in five controls to exclude structural cardiac anomalies; none of these had evidence of right-to-left shunting.

In all 31 patients cultures of tracheal aspirates were routinely performed every 3 days. The study was conducted according to the principles established in the Declaration of Helsinki and approved by the Medical Ethical Committee of our hospital.

### Study design

Of each patient the following data were recorded: gestational age, birth weight, diagnosis, survival, age at admission and at discharge or death, age at start of respiratory insufficiency, duration of ECMO and/or artificial ventilation, and duration of supplemental $O_2$ therapy. Outcome parameters were: mortality and $O_2$-dependency at 28 days, as a criterion for chronic lung disease.[19]

Bronchoalveolar lavage was performed on day 1, day 3 and day 7 and once every week thereafter as long as endotracheal intubation was continued and the child remained in our Intensive Care Unit. However, an unstable clinical condition was a reason to modify this schedule.

The following data were recorded at each BAL procedure: clinical events such as surgery, mean airway pressure (MAP), oxygenation index (OI), and $AaDO_2$.[16] Arterial blood gases were obtained within 6 hours of the time of BAL.

### BAL procedure

The procedure was performed directly after routine endotracheal suctioning by the nursing staff using a technique described by Grigg *et al.*[20] The patient's head turned left, a 5 Fr open-ended catheter (outer diameter 1.7 mm; Sherwood Medical, Petit Rechain, Belgium) was passed down the endotracheal tube and placed in wedge position. Warmed 0.9% NaCl was instilled in two aliquots of 1 ml/kg each. Gentle manual suctioning with a 20-ml syringe was directly performed after each aliquot. In most cases the ventilatory circuit was not broken. The whole procedure always took less than 1 min. The recovered fluid was immediately processed; 20 µl was aspirated for cell counting, the remaining fluid was centrifuged at $900 \times \boldsymbol{g}$. The supernatant was frozen at −80°C, the cells were resuspended in 0.9% NaCl and processed for cytocentrifugation.

### Measurements in BAL fluid

BAL fluid was diluted in Türk stain (1:10) for cell counting in a Bürker-haemocytometer. Differential cell counts were carried out on cytospin slide preparations with May–Gruenwald–Giemsa stain. In the supernatant 6-keto-$PGF_{1\alpha}$ and $TxB_2$ were measured by radioimmunoassay as described previously, using standard prostaglandins from Sigma Co. (St Louis, MO, USA) and antibodies from Advanced Magnetics Inc. (Cambridge, MA, USA).[21] Elastase-$\alpha_1$-proteinase inhibitor-complex (E-$\alpha_1$-PI) was determined using a commercially available kit (PMN Elastase; Merck Immunoassay 11332; Merck, Germany). Albumin was measured by immunoprecipitation using N-antiserum against human albumin (Behring OSAL 14/15; Behring, France). Human albumin (20%) (CLB, Amsterdam, The Netherlands) was used to obtain standard curves. Total protein was measured as described by Lowry,[22] standard curves were obtained using Preciset 6 g/100 ml (Boehringer 125610; Boehringer, Germany).

### Data analysis

Data were expressed as median (range) unless stated otherwise. Because of a low yield of BAL fluid, not all measurements could be performed for all samples, which resulted in incomplete data. Ventilatory parameters were compared between groups using the non-parametric Mann–Whitney U-test. Two tailed Spearman's rank correlation coefficient was used to study the relationship between different parameters in BAL fluid and ventilatory parameters during the first 3 days of treatment. In those cases where more than one BAL procedure was performed within the first 3 days, the average value of each parameter was used for calculation of the correlation coefficient. Statistical significance was assumed at the 5% level.

### Results

### Patient characteristics

The characteristics of all patients are summarized in Table 1. Ventilated controls had the following diagnoses: meconium peritonitis (n = 4), oesophageal atresia (n = 4), M. Hirschsprung, necrotizing enterocolitis, vesical exstrophy, hiatus hernia with Ehlers–Danlos syndrome, wet lung (each n = 1). None of the controls showed evidence of PPH at any time. Ten CDH-CV patients had left-sided CDH and three had a right-sided defect. Six CDH-CV patients had PPH: two of those, who never met the ECMO entry criteria as a result of a persistently low $PaO_2$, died during the first hours after birth. Four CDH-CV patients were $O_2$-dependent at the age of 28 days (one patient without documented PPH). Of the CDH-ECMO patients, who all had documented PPH, one had a bilateral diaphragmatic defect, the other four had left-sided CDH. Two survivors were operated on while undergoing ECMO, and they were both $O_2$-dependent at 28 days. The other three ECMO patients died from recurrent episodes of therapy-resistant pulmonary hypertension 5–11 days after decanulation; one of these patients was operated on during ECMO on day 20. In these three patients BAL was not performed after decanulation because their clinical condition was unstable and deteriorated rapidly.

In only one CDH-CV patient there was evidence of PPH during the BAL procedure; in all other patients the pre- and postductal transcutaneous $O_2$-saturation measurements were similar during BAL.

### Prostanoid concentrations in BAL fluid of CDH patients

In all but one patient the prostanoid concentration in BAL fluid could be measured at least

**Table 1**. Characteristics of control subjects and CDH patients

| | Controls n = 13 (13)[a] | CDH-CV n = 13 (11)[a] | CDH-ECMO n = 5 (2)[a] |
|---|---|---|---|
| Gestational age (weeks) | 38 (36–41) | 38.5 (34–41) | 39 (34–42) |
| Birth weight (grams) | 2710 (2350–3910) | 3140 (1550–4340) | 3000 (2380–3630) |
| Ventilation (days)[b] | 3 (1–5) | 10 (3–33) | 42.5 (34–51) |
| Supplemental $O_2$ (days)[b] | 4 (1–12) | 15 (3–47) | 58 (35–81)[f] |
| $O_2$-dependency at 28 days (n)[b] | 0 | 4 | 2 |
| Age at surgery CDH (days)[b] | | 4 (2–28[c]) | 9.5 (6–13) |
| Age at start of ECMO (hours) | | ND | 16 (6–42) |
| Duration of ECMO (days) | | ND | 14 (6–25) |
| MAP[d] (day 1–2) | 10 (7.7–25) | 20 (4.6–62)[e] | ND |
| (day 3–5) | 8.9 (5.1–19) | 17.7 (9.7–25)[e] | ND |
| OI[d] (day 1–2) | 3.3 (2.3–13) | 5.5 (1–250) | ND |
| (day 3–5) | 1.9 (1.1–4.8) | 3.9 (2.6–13) | ND |
| A-a$DO_2$[d] (day 1–2) | 51 (81–230) | 110 (17–638) | ND |
| (day 3–5) | 26 (18–81) | 64 (24–235) | ND |

The median (range) values are shown for different patient characteristics. CDH-CV, conventionally ventilated CDH patients; CDH-ECMO, ECMO-treated CDH patients. ND, not determined.
[a]The total number of patients per group is shown, the number of survivors is shown in brackets.
[b]Only data from survivors are shown.
[c]One child was diagnosed at the age of 27 days, surgery was performed one day after diagnosis, no documented PPH.
[d]Parameters calculated only at the time that BAL was performed, day 1–2: controls n = 6, CDH-CV n = 9; day 3–5: n = 6 for both groups.
[e]Significantly different from controls, $P < 0.05$.
[f]One child received additional $O_2$-therapy 1 month later and is still $O_2$-dependent at 32 months of age.

once; the sample volume of one CDH-CV patient, who died within several hours after birth, was too small to measure prostanoid concentrations. The concentrations of 6-keto-PGF$_{1\alpha}$ and TxB$_2$ are shown in Figs 1 and 2, respectively and in Table 2. Once CDH-CV patient with evidence of PPH at the time of BAL who died during the first day of life had a very high level of 6-keto-PGF$_{1\alpha}$, the same was true for the initial levels of two ECMO-treated CDH patients who died later (Fig. 1A). In the group of CDH survivors, only one patient who needed artificial ventilation for 4 weeks showed high levels of 6-keto-PGF$_{1\alpha}$, whereas all other CDH-CV and CDH-ECMO survivors had 6-keto-PGF$_{1\alpha}$ levels within the control ranges (Fig. 1B).

The concentrations of TxB$_2$ varied irrespective of outcome (Fig. 2). Deterioration of the clinical condition in one patient with PPH who developed sepsis on day 10, followed by thrombosis of the inferior vena cava, was not reflected by an increase in prostanoids initially, but by an increase in TxB$_2$ a few days later; 6-keto-PGF$_{1\alpha}$ remained low.

Surprisingly, in two infants who died, one CDH-CV patient and one CDH-ECMO patient,

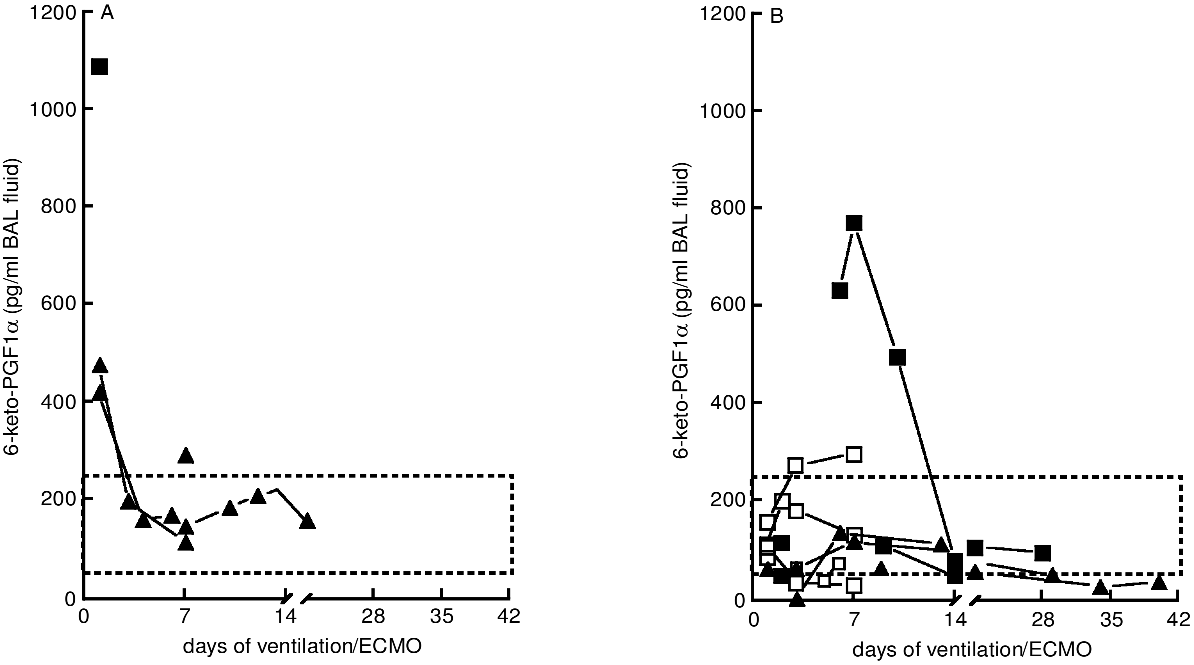

FIG. 1. The concentrations of 6-keto-PGF$_{1\alpha}$ in BAL fluid of non-surviving CDH patients (A) and CDH survivors (B) are shown. Conventionally ventilated patients, ■, □; ECMO-treated patients, △, ▲. Closed symbols represent a poor clinical outcome: death (A) or O$_2$-dependency at 28 days (B). Each measurement is indicated by an individual symbol, a line through the symbols indicates that more BAL procedures were performed on one patient. The box represents the interquartile range concentration of 6-keto-PGF$_{1\alpha}$ during the first 2 days of ventilation in 10 control patients without PPH.

**Table 2.** Prostanoids in BAL fluid of ventilated controls and CDH patients, and in CDH patients treated with ECMO

| | | Controls-CV[a,b] | CDH-CV[a,c] | CDH-ECMO[d] |
|---|---|---|---|---|
| 6-keto-PGF$_{1\alpha}$[e] | Day 1–2 | 73 (54–283) | 114 (12–1089) | 419 (61–455) |
| | Day 3–5 | 72 (21–107) | 64 (29–273) | 73 (0–162) |
| | Day 6–12 | ND | 120 (28–632) | 134 (28–632) |
| TxB$_2$[e] | Day 1–2 | 159 (91–252) | 153 (54–563) | 493 (360–626) |
| | Day 3–5 | 109 (54–780) | 153 (37–355) | 159 (0–458) |
| | Day 6–12 | ND | 467 (55–1172) | 177 (55–285) |

Values are indicated as median (range). Statistical analysis between the groups was not performed because of the missing data and the different time points. ND, not determined.
[a]CV, conventional ventilation.
[b]n = 9–12 on Day 1–2 and n = 6 on day 3 (no lavage data were obtained in controls after Day 3).
[c]n = 7–9 on Day 1–2 and n = 5–7 on day 3–5.
[d]n = 3 on Day 1–2 and n = 5 on Day 3–5.
[e]Concentrations are expressed as pg per ml bronchoalveolar lavage fluid.

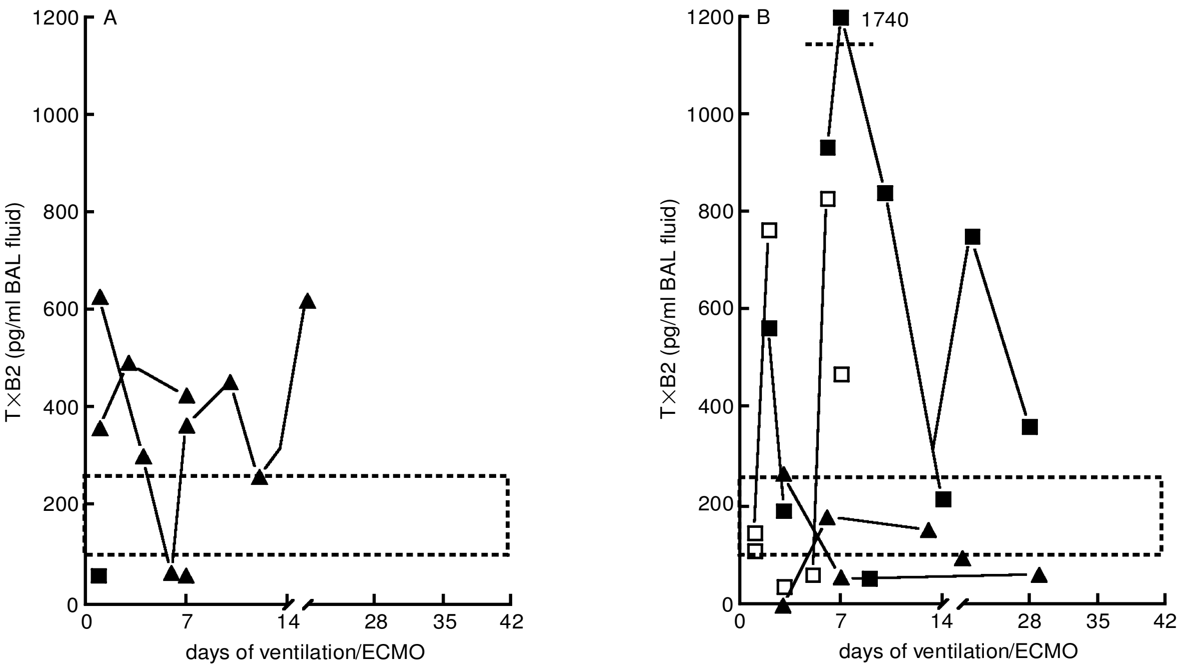

FIG. 2. The concentrations of $TxB_2$ in BAL fluid of non-surviving CDH patients (A) and CDH survivors (B) are shown. Conventionally ventilated patients, ■, □; ECMO-treated patients, △, ▲. Closed symbols represent a poor clinical outcome: death (A) or $O_2$-dependency at 28 days (B). Each measurement is indicated by an individual symbol, a line through the symbols indicates that more BAL procedures were performed on one patient. The box represents the interquartile range concentration of $TxB_2$ during the first 2 days of ventilation in 10 control patients without PPH.

the ratio of 6-keto-$PGF_{1\alpha}$ to $TxB_2$ was high: 20 and 5.2 respectively. The median ratio of 6-keto-$PGF_{1\alpha}$ to $TxB_2$ of the CDH-CV patients with PPH did not differ from that of those without PPH: 0.52 (0.14–20) versus 0.51 (0.02–2.5), respectively; controls without PPH had a median ratio of 0.6 (0.14–1.6). In the five CDH-ECMO patients, who all had PPH, the ratio of 6-keto-$PGF_{1\alpha}$ to $TxB_2$ was 0.7 (0.23–5.2).

The prostanoid concentrations did not correlate with any of the ventilatory parameters in the conventionally ventilated groups.

### Inflammatory markers in BAL fluid and correlation between different parameters

Cultures of tracheal aspirates were negative in all patients at the time of BAL. The total cell count, the percentage of neutrophilic granulocytes and macrophages, and the concentrations of protein, albumin, and E-$\alpha_1$-PI in BAL are shown in Table 3. During the first days the median cell count and the levels of protein, albumin, and E-$\alpha_1$-PI were high in the CDH-ECMO group.

In CDH-CV patients a negative correlation was found between the total cell count and MAP, OI, and $AaDO_2$ ($r = -0{\cdot}06$, $-0.58$, and $-0.56$ respectively; $P \leqslant 0{\cdot}05$; $n = 11$) and a positive correlation between % neutrophilic granulocytes and OI and $AaDO_2$ ($r = 0{\cdot}49$ and $0.57$, respectively; $P \leqslant 0{\cdot}05$; $n = 12$) during the first 3 days. In controls, albumin correlated positively with MAP and OI ($r = 0{\cdot}68$ and $0.69$, respectively; $P \leqslant 0{\cdot}05$; $n = 13$ and $8$, respectively), and total protein correlated positively with MAP ($r = 0{\cdot}63$; $P = 0{\cdot}01$; $n = 13$).

### Discussion

We have found different concentrations of prostanoids in BAL fluid of CDH patients with PPH: in infants who died and in one infant ventilated for more than 4 weeks either high levels of 6-keto-$PGF_{1\alpha}$ and $TxB_2$ compared to controls, or high levels of 6-keto-$PGF_{1\alpha}$ only. The ratio of 6-keto-$PGF_{1\alpha}$ to $TxB_2$ was high in two CDH patients who died. Survivors with evidence of PPH treated with ECMO, and CDH patients with $O_2$-dependency at 28 days had prostanoid levels which could not be discriminated from the levels of the other CDH patients. The levels of $TxB_2$ were variable in all CDH patients.

Increased levels of $TxB_2$ and 6-keto-$PGF_{1\alpha}$ have been reported in plasma of neonates with PPH who were treated with conventional ventilation or with ECMO.[6,7,9–12] Dobyns *et al.*

**Table 3**. Total cell count, cell differentiation, albumin, total protein and elastase-$\alpha_1$-proteinase inhibitor complex (E-$\alpha_1$-PI) in BAL fluid of ventilated controls and CDH patients, and in CDH patients treated with ECMO

| | | Controls-CV[a,b] | CDH-CV[a,c] | CDH-ECMO[d] |
|---|---|---|---|---|
| Cells ($\times 10^4$)[e] | Day 1–2 | 8.7 (1.7–90) | 3.5 (1.7–63) | 186 (28–227) |
| | Day 3–5 | 13 (1.7–38) | 10 (1.7–38) | 43 (23–69) |
| | Day 6–12 | ND | 12 (1.7–19) | 30 (1.7–59) |
| Neutrophils (%) | Day 1–2 | 70 (2–90) | 73 (10–85) | 79 (36–93) |
| | Day 3–5 | 26 (11–86) | 35 (3–72) | 53 (33–86) |
| | Day 6–12 | ND | 55 (17–76) | 5 (0–26) |
| Macrophages (%) | Day 1–2 | 22 (8–87) | 12 (2–82) | 14 (3–55) |
| | Day 3–5 | 61 (8–84) | 60 (13–92) | 46 (9–63) |
| | Day 6–12 | ND | 32 (20–50) | 89 (74–99) |
| Albumin (mg)[e] | Day 1–2 | 0.1 (0.005–0.31) | 0.1 (0.04–1.11) | 0.29 (0.02–1.5) |
| | Day 3–5 | 0.09 (0.03–0.16) | 0.05 (0.03–0.15) | 0.11 (0.02–0.6) |
| | Day 6–12 | ND | 0.06 (0.01–0.24) | 0.02 (0.02–0.06) |
| Protein (mg)[e] | Day 1–2 | 0.31 (0.02–1.67) | 0.26 (0.12–2.03) | 1.48 (0.09–2.9) |
| | Day 3–5 | 0.25 (0.1–0.42) | 0.16 (0.08–0.34) | 0.41 (0.26–4.14) |
| | Day 6–12 | ND | 0.13 (0.06–0.66) | 0.12 (0.03–0.19) |
| E-$\alpha_1$-PI ($\mu$g)[e] | Day 1–2 | 0.04 (0–1.95) | 0.03 (0–0.63) | 0.24 (0.004–0.9) |
| | Day 3–5 | 0.04 (0–0.21) | 0.03 (0–0.07) | 0.07 (0–0.57) |
| | Day 6–12 | ND | 0.02 (0–0.15) | 0.02 (0.003–0.09) |

Values are indicated as median (range). Statistical analysis between the groups was not performed because of the missing data and the different time points. ND, not determined.
[a]CV, conventional ventilation.
[b]n = 9–12 on Day 1–2 and n = 6 on Day 3–5 (no lavage data were obtained in controls after Day 3).
[c]n = 7–9 on Day 1–2, n = 5–7 on Day 3–5, and n = 4–6 on Day 6–12.
[d]n = 3 on Day 1–2, n = 5 on Day 3–5, and n = 3–4 on Day 6–12.
[e]Concentrations are expressed per ml bronchoalveolar lavage fluid.

described increased levels of $TxB_2$, 6-keto-$PGF_{1\alpha}$, $PGD_2$, $PGE_2$, $LTB_4$ and $LTE_4$ in BAL fluid of neonates with PPH.[8] Prostanoid levels in plasma and in BAL fluid were shown to decrease during the course of treatment in these children[7,8] and in one CDH patient treated with ECMO.[11] Dobyns *et al.* found persisting high levels of 6-keto-$PGF_{1\alpha}$ and $TxB_2$ in BAL fluid of ECMO-treated children with PPH and a poor outcome, whereas the prostanoid levels decreased rapidly in PPH patients with a good outcome.[8] Our findings of high levels of 6-keto-$PGF_{1\alpha}$ in infants who died are in accordance with these findings, but the variable $TxB_2$ levels in all CDH patients – irrespective their clinical outcome – contradict their observations.

To determine whether the high levels of prostanoids in BAL fluid of some CDH patients with PPH are a specific feature of the abnormal pulmonary vasculature in CDH, these levels should be compared with prostanoid levels in BAL fluid of children without CDH who need ventilatory support to the same extent as the most severely ill CDH patients. Our control population consisted of infants who had mild ventilatory requirements as reflected by the ventilatory parameters. Therefore, these controls did not allow for such comparison.

The relatively high and variable $TxB_2$ levels in the CDH patients, irrespective of the outcome, suggest that the clinical situation in this group of patients is not adequately reflected by the $TxB_2$ concentrations in BAL fluid. An earlier study from our group showed a correlation between plasma prostanoid levels and ventilatory parameters in CDH patients.[12] We were not able to demonstrate such correlations in BAL in our study. This may indicate that prostanoid levels in the pulmonary vasculature are not adequately reflected in BAL fluid, as has been suggested by Abman *et al.*[23] This was supported by our finding that in non-ventilated neonatal rats with CDH the concentration of $TxB_2$ is 10-fold higher in lung tissue than in BAL fluid.[24] However, in these rat pups the ratio of 6-keto-$PGF_{1\alpha}$ to $TxB_2$ was consistently increased in lung tissue and in BAL fluid compared with controls directly after birth, suggesting that this parameter in BAL fluid may be representative for the values in lung tissue.[24] In the present study a similar observation was made in two CDH patients, but this was not a consistent finding for all patients with a poor outcome.

The increased ratios of 6-keto-$PGF_{1\alpha}$ to $TxB_2$ suggest that the lungs compensate maximally for the pulmonary vasoconstriction in CDH. It is noteworthy that in neonatal rats with CDH the presence of more pulmonary neuroendocrine cells containing calcitonin gene-related peptide, a peptide with a known vasodilatory activity, has been reported.[25–27] Furthermore, in foetal lungs of rat pups with CDH, increased mRNA levels of endothelin have been observed recently (T. Okazaki, personal communication). Interestingly, Bos *et al.* reported that in CDH patients, all non-respondents to intravenous prostacyclin therapy died.[28] We speculate that in some CDH patients the lungs already compensate maximally for pulmonary vasoconstriction and may be insensitive to therapeutic interventions with pulmonary vasodilators, such as prostacyclin and nitric oxide.

Increased cell counts, neutrophil numbers, albumin, and elastase activity have been reported in tracheal aspirates or BAL fluid of prematurely born ventilated patients who developed chronic lung disease.[13–15] in the present study, concentrations of cells, protein, albumin, and E-$\alpha_1$-PI complex in the BAL fluid of CDH-ECMO patients were high, compared with conventionally ventilated CDH patients and controls during the first days of treatment. To our knowledge these parameters have not been reported before in lung lavages of ECMO-treated neonates. We assume that increased vascular permeability with an influx of cells is responsible and related to lung injury and activation of the inflammatory cascade before ECMO, or to neutrophil activation and protein influx as part of the capillary leakage syndrome in the initial phase of ECMO.[29] Our study does not allow for definite conclusions regarding the question of whether the high cell counts, and the high levels of protein, albumin, and elastase-$\alpha_1$-PI-complex in ECMO-treated CDH patients result from the disease state or from the ECMO procedure.

In conclusion, we found that in some CDH patients with PPH high prostanoid concentrations in BAL fluid were associated with a poor outcome. These patients died within a few hours of birth, or from recurrent episodes of therapy-resistant pulmonary hypertension several days after ECMO. The high levels of 6-keto-$PGF_{1\alpha}$ might have been induced by the hypoxic vasoconstriction in these patients[30] or reflect an attempt by the lungs to compensate maximally for the pulmonary vasoconstriction. The variation in $TxB_2$ concentrations may reflect lung injury and increased vascular permeability. This study illustrates the difficulties of performing BAL procedures on a regular basis in severely ill neonates and interpreting the prostanoid levels in BAL fluid. Based on our findings we feel that the predictive value of prostanoid levels in BAL fluid with respect to outcome is questionable.

### References

1. Molenaar JC, Bos AP, Hazebroek FWJ, Tibboel D. Congenital diaphragmatic hernia, what defect? *J Pediatr Surg* 1991; **26**: 248–254.
2. Langham MR Jr, Kays DW, Ledbetter DJ, Frentzen B, Sanford LL, Richards DS. Congenital diaphragmatic hernia. Epidemiology and outcome. *Clin Perinatol* 1996; **23**: 671–689.
3. Heiss KF, Clark RH. Prediction of mortality in neonates with congenital diaphragmatic hernia treated with extracorporeal membrane oxygenation. *Crit Care Med* 1995; **23**: 1915–1919.
4. Moncada S, Vane JR. Pharmacology and endogenous roles of prostaglandin endoperoxides, thromboxane $A_2$, and prostacyclin. *Pharmacol Rev* 1979; **30**: 293–331.
5. Stenmark KR, James SL, Voelkel NF, Toews WH, Reeves JT, Murphy RC. Leukotriene $C_4$ and $D_4$ in neonates with hypoxemia and pulmonary hypertension. *N Engl J Med* 1983; **309**: 77–80.
6. Hammerman C, Lass N, Strates E, Komar K, Bui KC. Prostanoids in neonates with persistent pulmonary hypertension. *J Pediatr* 1987; **110**: 470–472.
7. Bui KC, Hammerman C, Hirschl R, *et al.* Plasma prostanoids in neonatal extracorporeal membrane oxygenation. *J Thorac Cardiovasc Surg* 1991; **101**: 612–617.
8. Dobyns EL, Westcott JY, Kennaugh JM, Ross MN, Stenmark KR. Eicosanoids decrease with successful extracorporeal membrane oxygenation therapy in neonatal pulmonary hypertension. *Am J Respir Crit Care Med* 1994; **149**: 873–880.
9. Nakayama DK, Motoyama EK, Evans R, Hanankan C. Relation between arterial hypoxemia and plasma eicosanoids in neonates with congenital diaphragmatic hernia. *J Surg Res* 1992; **53**: 615–620.
10. Ford WDA, James MJ, Walsh JA. Congenital diaphragmatic hernia: association between pulmonary vascular resistance and plasma thromboxane concentrations. *Arch Dis Child* 1984; **59**: 143–146.
11. Stolar CJH, Dillon PW, Stalcup SA. Extracorporeal membrane oxygenation and congenital diaphragmatic hernia: modification of the pulmonary vasoactive profile. *J Pediatr Surg* 1985; **20**: 681–683.
12. Bos AP, Tibboel D, Hazebroek FWJ, Stijnen T, Molenaar JC. Congenital diaphragmatic hernia: impact of prostanoids in the perioperative period. *Arch Dis Child* 1990; **65**: 994–995.
13. Merritt TA, Cochrane CG, Holcomb K, *et al.* Elastase and $\alpha_1$-proteinase inhibitor activity in tracheal aspirates during respiratory distress syndrome. *J Clin Invest* 1983; **72**: 656–666.
14. Ogden BE, Murphy SA, Saunders GC, Pathak D, Johnson JD. Neonatal lung neutrophils and elastase/proteinase inhibitor balance. *Am Rev Respir Dis* 1984; **130**: 817–821.
15. Groneck P, Götze-Speer B, Opperman M, Eiffert H, Speer CP. Association of pulmonary inflammation and increased microvascular permeability during the development of bronchopulmonary dysplasia: a sequential analysis of inflammatory mediators in respiratory fluids of high-risk preterm neonates. *Pediatrics* 1994; **93**: 712–718.
16. Hazebroek FWJ, Tibboel D, Bos AP, *et al.* Congenital diaphragmatic hernia: impact of preoperative stabilization. A prospective pilot study in 13 patients. *J Pediatr Surg* 1988; **23**: 1139–1146.
17. Milerad J, Walsh WF. Commentary on neonatal ECMO: a North American and Scandinavian perspective. *Acta Paediatr* 1995; **84**: 841–847.
18. Klein MD, Whittlesey GC. Extracorporeal membrane oxygenation. *Pediatr Clin North Am* 1994; **41**: 365–384.
19. Bancalari E, Gerhardt T. Bronchopulmonary dysplasia. *Pediatr Clin North Am* 1986; **33**: 1–23.
20. Grigg J, Arnon S, Silverman M. Fractional processing of sequential bronchoalveolar lavage fluid from intubated babies. *Eur Respir J* 1992; **5**: 727–732.
21. Zijlstra FJ, Vincent JE, Mol WM, Hoogsteden HC, Van Hal PT, Jongejan RC. Eicosanoid levels in bronchoalveolar lavage fluid of young female smokers and non-smokers. *Eur J Clin Invest* 1992; **22**: 301–306.
22. Lowry OH, Rosebrough NJ, Farr AL, Randall RJ. Protein measurement with the Folin phenol reagent. *J Biol Chem* 1951; **193**: 265–275.
23. Abman SH, Stenmark KR. Changes in lung eicosanoid content during normal and abnormal tranistion in perinatal lambs. *Am J Physiol* 1992; **262**: L214–222.
24. IJsselstijn H, Zijlstra FJ, van Dijk JPM, de Jongste JC, Tibboel D. Lung eicosanoids in perinatal rats with congenital diaphragmatic hernia. *Mediators of Inflammation* 1997; **6**: 39–45.
25. IJsselstijn H, Perrin DG, de Jongste JC, Cutz E, Tibboel D. *J Pediatr Surg* 1995; **30**: 413–415.
26. Yamataka T, Puri P. Increased intracellular levels of calcitonin gene-related peptide-like immunoreactivity in pulmonary endocrine cells in an experimental model of congenital diaphragmatic hernia. *Pediatr Surg Int* 1996; **11**: 448–452.
27. Brain SD, Williams TJ, Tippins JR, Morris HR, MacIntyre I. Calcitonin gene-related peptide is a potent vasodilator. *Nature* 1985; **313**: 54–56.
28. Bos AP, Tibboel D, Koot VCM, Hazebroek FWJ, Molenaar JC. Persistent pulmonary hypertension in high-risk congenital diaphragmatic hernia patients: incidence and vasodilator therapy. *J Pediatr Surg* 1993; **28**: 1463–1465.

29. Fortenberry JD, Bhardwaj V, Niemer P, Cornish JD, Wright JA, Bland L. Neutrophil and cytokine activation with neonatal extracorporeal membrane oxygenation. *J Pediatr* 1996; **128**: 670–678.
30. Weir EK, McMurthy IF, Tucker A, Reeves JT, Grover RF. Prostaglandin synthetase inhibitors do not decrease hypoxic pulmonary vaso-constriction. *J Appl Physiol* 1976; **41**: 714–718.

ACKNOWLEDGEMENTS. This work was financially supported by the Netherlands Asthma Fund, project number 91.56.

The authors want to thank the following for technical assistance: Ms J. van Dijk and C. Tak from the Department of Pharmacology (EUR), Ms I. Dekker from the Specialized Hematological Laboratory (head: Dr K. Hählen), Ms J. van't Hoff from the Clinical Biochemical Laboratory (head: Dr. G.J.M. Boerma) in our hospital, and P. van Schalkwijk from the Biochemical Laboratory from the EDC (EUR). Thanks are also due to the medical staff and nursing staff of the Paediatric Surgical Intensive Care Unit of our hospital for assistance during the BAL procedures.

**Received 4 April 1997; accepted 17 April 1997**
